# The Impact of the *Matricaria chamomilla* L. Extract, Starch Solution and the Photoinitiator on Physiochemical Properties of Acrylic Hydrogels

**DOI:** 10.3390/ma15082837

**Published:** 2022-04-12

**Authors:** Mateusz Jamroży, Magdalena Głąb, Sonia Kudłacik-Kramarczyk, Anna Drabczyk, Paweł Gajda, Bożena Tyliszczak

**Affiliations:** 1 Department of Materials Science, Faculty of Materials Engineering and Physics, Cracow University of Technology, 37 Jana Pawła II Av., 31-864 Krakow, Poland; sonia.kudlacik-kramarczyk@pk.edu.pl (S.K.-K.); anna.drabczyk2@pk.edu.pl (A.D.); bozena.tyliszczak@pk.edu.pl (B.T.); 2 Department of Sustainable Energy Development, Faculty of Energy and Fuels, AGH University of Science and Technology, 30 Mickiewicza Av., 30-059 Krakow, Poland; pgajda@agh.edu.pl

**Keywords:** acrylic hydrogels, chamomile extract, starch, starch gelatinization, release profile, sorption capacity, tensile strength, simulated physiological liquids

## Abstract

*Matricaria chamomilla* L. extract is well-known for its therapeutic properties; thus, it shows potential to be used to modify materials designed for biomedical purposes. In this paper, acrylic hydrogels modified with this extract were prepared. The other modifier was starch introduced into the hydrogel matrix in two forms: room-temperature solution and elevated-temperature solution. Such hydrogels were synthesized via UV radiation, while two types of photoinitiator were used: 2-hydroxy-2-methylpropiophenone or phenylbis(2,4,6-trimethylbenzoyl) phosphine oxide. The main task of performed research was to verify the impact of particular modifiers and photoinitiator on physicochemical properties of hydrogels. Studies involved determining their swelling ability, elasticity, chemical structure via FTIR spectroscopy and surface morphology via the SEM technique. Incubation of hydrogels in simulated physiological liquids, studies on the release of chamomile extract from their matrix and their biological analysis via MTT assay were also performed. It was demonstrated that all investigated variables affected the physicochemical properties of hydrogels. The modification of hydrogels with chamomile extract reduced their absorbency, decreased their thermal stability and increased the cell viability incubated with this material by 15%. Next, hydrogels obtained by using phenylbis(2,4,6-trimethylbenzoyl) phosphine oxide as a photoinitiator showed lower absorbency, more compact structure, better stability in SBF and a more effective release of chamomile extract compared to the materials prepared by using 2-hydroxy-2-methylpropiophenone. It was proved that, by applying adequate reagents, including both photoinitiator and modifiers, it is possible to obtain hydrogels with variable properties that will positively affect their application potential.

## 1. Introduction

Chamomile (*Matricaria chamomilla* L.) is a well-known species of a medicinal plant that derives from the Asteraceae family. It has been used for years as a medicinal substance both in folk and traditional medicine [1,2]. Dried chamomile flowers contain many terpenoids and flavonoids, which enhance this plant with therapeutic properties. Thus the chamomile-based preparations are widely applied in the case of such ailments as inflammations, ulcers, gastrointestinal disorders, rheumatic pains or hemorrhoids [3]. Furthermore, essential oils included in the chamomile extract are also widely applied in aromatherapy and cosmetics. The preparations based on this plant are used in various forms, including the medicinal preparation based on active substances extracted from its dried flowers (the most commonly used form), or in the form of powder from the flowers acting as a modifier of the ointments or gels [4,5]. Chamomile, as a component of numerous externally applied preparations, finds application in the treatment of inflammations of the skin and mucous membranes, as well as in the treatment of bacterial infections of the mouth and the gums [6]. On the other hand, the oral use of chamomile is mainly associated with its sedative effect and use in the treatment of insomnia [7].

Among the healing properties of chamomile, special attention is paid to its anti-inflammatory properties. The studies on these properties were performed, for example, by Fabian et al. The research described in this work was focused on reducing inflammation and paw swelling in mice. They received the chamomile oil in various concentrations. It was proved that the parameters of the experimental inflammatory models in mice improved depending on the applied concentration of the chamomile oil [8]. The effectiveness of the application of the chamomile extract as an anti-inflammatory agent was confirmed by the investigations performed by Bhaskaran et al. [9]. Based on the results obtained, it was concluded that chamomile shows a clear inhibitory effect on the expression of the iNOS gene; thus, it plays an important role in mediating inflammatory responses. Subsequently, it was proved that chamomile works on the basis of a similar mechanism of action as that of non-steroidal inflammatory drugs, i.e., by inhibiting the activity of the COX-2 enzyme [10]. In another work, Sakai et al. [11] showed the anti-inflammatory effect of sodium azulene sulfonate, which is a water-soluble derivative of azulene, which, in turn, is an anti-inflammatory component of chamomile. Such an anti-inflammatory effect was confirmed against the pharyngitis model.

Apart from the anti-inflammatory properties of chamomile, its antibacterial and antioxidant activity are also promising in terms of its potential use. The effect of the chamomile flowers on mouse skin infection was also investigated by El-Shouny et al. [12]. In this work, the chamomile extract was analyzed in terms of its antimicrobial activity against *Staphylococcus aureus* (Gram-positive bacteria) and the strain of *Candida albicans*. As a result of the performed research, it was proved that the tested material showed both antibacterial activity against *S. aureus* and antifungal activity against *C. albicans*. Moreover, the antibacterial effect of chamomile was also demonstrated in other investigations [13,14].

In terms of the antibacterial and anti-inflammatory properties of chamomile, numerous investigations of the effectiveness of its topical application for wound-healing acceleration are being performed. For example, Nayak et al. [15] conducted studies on the impact of chamomile on the healing process, while the study was carried out both for the tested and control group (not treated with chamomile). It was proved that, in the tested group, after 15 days of the study, there was a greater reduction in the wound area compared to the wound area in the control group. Moreover, in the tested group, a faster epithelization, an increased rate of the wound contraction and a much higher breaking strength, as well as an increased content of hydroxyproline, were observed, indicating the high application potential of chamomile in regenerative processes. In addition, it was demonstrated that the healing in the presence of chamomile proceeded faster than in the presence of corticosteroids [16]. Furthermore, Kazemian et al. [17] showed that the antibacterial effect, as well as the wound regeneration, is significantly higher in the case of the application of chamomile-based ointments than in the case of tetracycline ointments. Topical applications of chamomile-based preparations concern mainly the preparations in the form of an ointment or a gel. When considering the possibility of accelerating the regenerative processes after the use of chamomile, the application of this substance as a modifier of hydrogels becomes a prospect. These materials are widely used in many fields of modern medicine, including tissue engineering, or for the preparation of controlled drug delivery systems and innovative third-generation dressing materials [18,19,20,21,22].

Due to the occurrence of the dependence between the composition of hydrogel materials and their properties, it is possible to develop materials with desirable properties. The type of the photoinitiator used may be mentioned among factors affecting the properties of hydrogel materials. This compound is of great importance during the photopolymerization and affects the structure of the hydrogels [23]. The dependence between the type of the photoinitiator and mechanical and rheological properties of hydrogels was demonstrated by Yang et al. [24], for example. Next, Pahoff et al. [25] proved that the photoinitiator influenced the biological properties of hydrogels. Moreover, hydrogel materials loaded with chondrocytes and reinforced with polycaprolactone-based microfibers were evaluated in terms of induced biological response. It was demonstrated that the type of photoinitiator significantly affects the physical and biochemical properties of hydrogels, as well as the viability of cells incubated in the presence of these materials. In turn, studies performed by Wiley et al. [26] demonstrated how the photopolymerization rate may be adjusted by the wavelength, irradiation intensity and the type of the photoinitiator, so as to control the properties of synthesized hydrogels. The base materials of the hydrogel matrix, such as the polymers, crosslinking agent and photoinitiator, also affect the properties of the obtained materials. Moreover, these properties strictly depend on the type of the modifier applied [27].

Thus, in this study, the main focus was on determining the impact of the type of the photoinitiator used for photopolymerization (two photoinitiators were used, i.e., phenylbis(2,4,6-trimethylbenzoyl) phosphine oxide and 2-hydroxy-2-methylpropiophenone; these compounds were selected due to the fact that one of their absorbency maximums appears at the wavelength 320 nm, which is within the wavelength range of the UV lamp used by us to perform the photopolymerization process), as well as the modifiers, such as *Matricaria chamomilla* L. extract and starch solution (the solution of this polysaccharide prepared via two various methodologies, i.e., at elevated and ambient temperature), on the physicochemical and biological properties of the hydrogels. In our previous research [28], the impact of different concentrations of cold-prepared starch on the properties of acrylic hydrogels was characterized. In terms of the continuation of this research topic, here the focus was on determining the impact of “hot” and “cold” starch with the same concentration on the properties of hydrogels. The combination of biocompatible hydrogel materials with chamomile extract providing antibacterial and anti-inflammatory properties leads to the preparation of dressing materials with favorable properties supporting regenerative processes. By determining the dependence between the amount of the modifier used or the type of the photoinitiator used and the properties of the hydrogels, it is possible to verify the compositions of hydrogels which are more or less adequate for particular applications. For example, in some cases, the high swelling ability of a hydrogel is more desirable; in the other cases, the tensile strength is more important.

## 2. Materials and Methods

### 2.1. Materials

Potassium hydroxide (pure, p.a.) and corn starch (pure, p.a.) were bought from Avantor Performance Materials, Gliwice, Poland, S.A. Acrylic acid (99%; p.a., anhydrous, contains 200 ppm MEHQ as inhibitor), diacrylate poly(ethylene glycol) (crosslinking agent; average molecular weight Mn = 575 g/mol; density = 1.12 g/mL), 2-hydroxy-2-methylpropiophenone (photoinitiator, density: 1.077 g/mL) and phenylbis(2,4,6-trimethylbenzoyl) phosphine oxide were received from Merck (Darmstadt, Germany). *Matricaria chamomilla* L. leaves were purchased in Herbapol (Lublin, Poland).

### 2.2. Preparation of Matricaria chamomilla L. Extract

**Figure 1:**
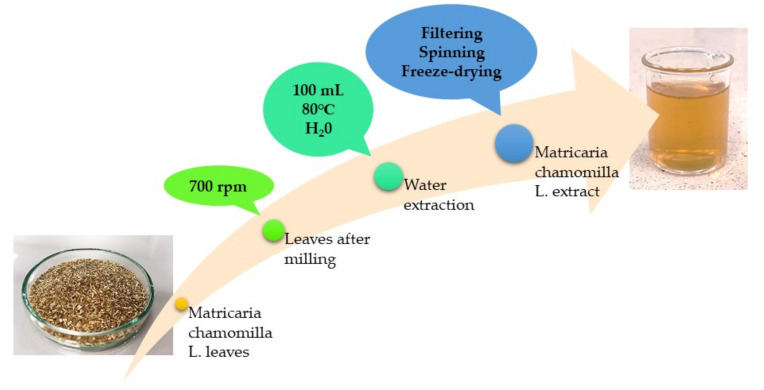
Scheme of the preparation of the *Matricaria chamomilla* L. extract.

In order to prepare *Matricaria chamomilla* L. extract for the modification of hydrogels, 20 g of plant leaves was ground, using a mill (MRC Ltd.-Laboratory Equipment, Holon, Israel). The obtained powder was then treated with 100 mL of water, and the extraction process was performed for 15 min, at 80 °C. The prepared infusion was next filtered. The filtrate obtained was centrifuged (15 min; 2700 rpm), the supernatant was decanted and the precipitate was subjected to the lyophilization process. The prepared powder was stored at ambient temperature in a dry and shady place. The applied procedure is presented schematically in Figure 1.

Finally, in order to prepare the extract, 2.0 g of previously lyophilized powder was introduced into distilled water (50 mL), and the whole was boiled for 15 min. Such prepared extract was used next as a modifier of hydrogel materials.

### 2.3. Synthesis of Hydrogel Polymers

Firstly, acrylic acid was neutralized by using an adequate amount of 40% KOH solution. Next, the starch solution was added to the neutralized acid, while one half of samples were prepared by using the solution of this polysaccharide at ambient temperature (defined as “cold” starch), and the second half by using starch solution at 60 °C (defined as “hot” starch). Next, the prepared reaction mixtures (consisting of acrylic acid and starch solution) were supplemented with adequate amounts of chamomile extract, crosslinking agent (diacrylate poly(ethylene glycol)) and photoinitiator (one series with 2-hydroxy-2-methylpropiophenone (Darocur 1173), and the second one with phenylbis(2,4,6-trimethylbenzoyl) phosphine oxide). The amounts of the photoinitiator and crosslinking agent applied were selected based on our previously performed experiments [28]. Detailed compositions of all hydrogels are presented below, in Table 1.

In Table 2, the designations of prepared samples are presented.

**Figure 2:**
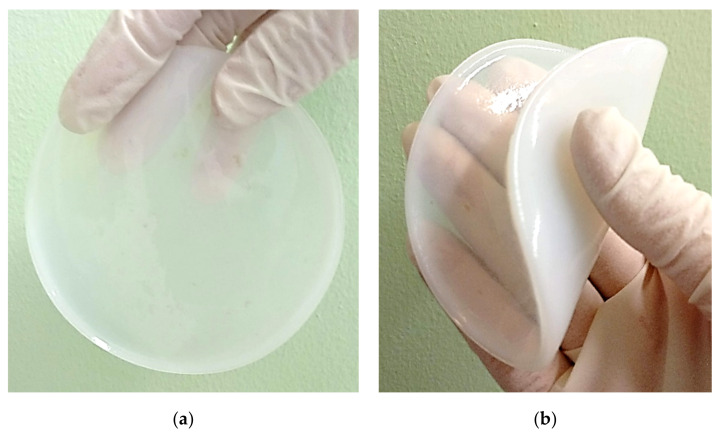
Example images of obtained materials, i.e., sample 5_cold_S/0_MC/Phot_A (**a**) and sample 5_cold_S/5_MC/Phot_A (**b**).

In Figure 2, example images of the obtained hydrogel materials are shown.

The prepared materials were subsequently subjected to investigations aimed at verifying their physicochemical properties, e.g., sorption capacity or tensile strength, as well as determining the release profile of the active substance from the hydrogel matrices.

### 2.4. Methodology of the Research

#### 2.4.1. Investigations on the Sorption Capacity of Hydrogels

The hydrogels obtained were developed for application as innovative wound dressings. One of the main roles of such dressings is wound exudate adsorption, enabling the wound-healing process. Thus, the first study performed involved determining the sorption properties of the hydrogels. The investigations were carried out in simulated physiological fluids, including SBF (simulated body fluid, isotonic to the plasma of human blood), hemoglobin (1% aqueous solution), Ringer liquid (infusion fluid) and distilled water. The study was performed as follows: Each hydrogel sample (weighing approximately 1.0 g) was added to the tested liquid (50 mL). After 1 h of the study, the hydrogels were separated from the tested media, weighed in a swollen state (after removing the excess water via a paper towel) and introduced to the tested liquids again to verify the swelling after 24 and 72 h, respectively (the procedure was the same after these time periods, i.e., separating from the liquid, removing the excess water and weighing). The sorption capacity of the samples was defined via a swelling ratio, Q, calculated by using the Equation (1):

(1) $$Q=\frac{m-m_{0}}{m_{0}}$$

where *Q* is the swelling ratio, g/g; m is the weight of hydrogel sample after swelling, g; and $m_{0}$ is the weight of dry sample (before swelling), g.

The research was conducted for all prepared hydrogels, and the main purpose was to verify the impact of the composition (including the type of the photoinitiator used, the form of starch solution used and the presence of chamomile extract) of tested hydrogels on their sorption ability.

#### 2.4.2. Assessment of the Mechanical Properties of Hydrogel Materials

Considering the application of the developed hydrogels as dressing materials, it is significant to know whether these materials are elastic or resistant to breakage under tension. The study was performed by using the Brookfield CT3 texture analyzer (Middleboro, MA, USA), and the procedure was carried out according to the ISO standards (ISO 37 type 2, ISO 527-2 type 5A). Before the analysis, a ZCP020 Cutting Press (ZwickRoell, Wrocław, Poland) was used to prepare paddle-shape samples (having the following dimensions: width—3.0 mm, length—30.0 mm and depth—1.5 mm). Such samples were next dried at ambient temperature, under pressure (to maintain their shape). Dry materials were fixed carefully within the texture analyzer (between its jaws). During the analysis, the jaws parting was observed, which proceeded simultaneously with the sample stretching. The study was performed until the sample was cracked. Such a procedure provided information concerning the elasticity of tested materials and their resistance to the tension applied.

#### 2.4.3. Morphological Analysis of Hydrogels via Scanning Electron Microscopy (SEM)

The next study involved characterizing the surface morphology of the hydrogels. The research was carried out via the use of a Jeol 5510LV (Jeol Ltd., Tokyo, Japan) Scanning Electron Microscope. Firstly, the samples were dried at ambient temperature and sputtered with gold. Next, they were subjected to the study, which was conducted at room temperature.

#### 2.4.4. Studies on the Release of the Active Substance from Developed Hydrogel Matrices

An important part of the performed investigations was also determining the release profile of the active substance—chamomile extract—from the developed materials. This is due to the fact that dressing materials with drug delivery function may effect positively the wound healing process and, thus, accelerate the wound regeneration. Studies were performed by using two different environments, i.e., an acidic environment (with pH = 2.0 providing by 2% citric acid solution) and an alkaline one (with pH = 7.4; phosphate buffer (PBS)) to verify the conditions in which the release proceeds more efficiently. The first task was to verify which substances from a rich composition of chamomile extract may be identified spectrophotometrically. Among all substances, polysaccharides were selected, due to the fact that they form colorful complexes with aluminum chloride; thus, their presence (and concentration) may be determined via UV–Vis spectrophotometry (UV–Vis Visible Spectrophotometer V-500 (Thermo Fisher Scientific, Waltham, MA, USA) was applied). The absorbance of chamomile extract used during the synthesis was defined as 100% (observed at a wavelength of 340 nm). The procedure of the study was as follows: Firstly, hydrogel samples were introduced into flasks containing 200 mL of tested environments (i.e., citric acid solution and PBS buffer). Then the flasks were placed in the shaking incubator (Hanchen ES-60E Temperature Controlled Incubator and Shaker Scientific Incu-Shaker Shaking Incubator, Tokyo, Japan) and shaken (rpm = 80). Importantly, the research was conducted at 36.6 °C, thereby imitating the temperature occurring in the human body. Next, after specific time periods, 3 mL of the tested solutions was taken and transferred into the cuvettes, which were supplemented additionally with 0.125 mL of AlCl_3_ solution (5% solution in methanol), and remained for 0.5 h. Next, the mixtures were analyzed spectrophotometrically, while the study was performed at ambient temperature. Importantly, after sampling, the flasks were supplemented with 3 mL of fresh environment, so as to keep the same volume of the tested release environment.

#### 2.4.5. Characterization of the Chemical Structure of Hydrogels via FTIR Technique

Fourier-transform infrared (FTIR) spectroscopy was performed to identify the presence of functional groups’ characteristics for the tested samples. Moreover, the samples were immersed for 30 days in simulated body fluid (SBF), and, after such an immersion, they were also subjected to the FTIR analysis to verify the impact of such an environment on their chemical structure. The study was performed at room temperature, while the following apparatus was applied: Thermo Scientific Nicolet iS5 equipped with ATR diamond accessory (Loughborough, UK). The spectra of hydrogels were recorded within the wavenumber range of 4000–500 cm^−1^ (resolution of 4.0 cm^−1^, 32 scans).

#### 2.4.6. Thermal Analysis of Hydrogel

The main purpose of the study was to determine the thermal stability of hydrogels, and most of the attention was paid to the impact of the introduced modifiers on the thermal properties of the tested polymers. The study was performed for hydrogel samples weighing 5.00 mg. The thermogravimetric analysis was conducted under air atmosphere in the temperature range of 20–600 °C, and the heating rate was 10 °C/min. The Netzsch TG 209 F1 Libra thermogravimetric analyzer (Netzsch, Selb, Germany) was applied for measurements.

#### 2.4.7. Studies on Cytotoxicity of Hydrogels via MTT Reduction Assay

The study was performed to verify the potential application of the developed hydrogels for biomedical purposes and constituted a preliminary research providing information as to whether the developed materials may be subjected to more advanced biological studies. The cytotoxicity of the hydrogels was determined via an MTT reduction assay. Here, the cell viability was defined via verifying the metabolic activity of the cell lines after exposure to the analyzed samples. The study involved defining the activity of the mitochondrial dehydrogenase to convert MTT reagent (soluble salt of tetrazol (3-(4,5-dimethylthiazol- 2-yl)-2,5-diphenyletrazol bromide)) to formazan (dark blue and insoluble product of this reaction). Such formed formazan crystals were subsequently dissolved in DMSO (or isopropanol), while the color intensity of the solution was verified via UV–Vis spectrophotometry (492–570 nm). The amount of the cells was then defined by the proportionality of the amount of the reduced MTT to the oxidative activity of the cellular mitochondria. In the research, L929 murine fibroblasts received from American Type Cell Culture Collection (Rockville, MD, USA) were applied. These cells were incubated in the culture flasks on RPMI-1640 medium containing penicillin (100 U/mL), streptomycin (100 μg/mL) and inactivated bovine serum (10 wt.%; Cytogen, Zgierz, Poland), under standard conditions (5% CO_2_, 37 °C, >90% humidity). Before the study, the suspension of L929 murine fibroblasts (concentration 2 × 10^5^ cells/mL) was prepared. Subsequently, the cell suspension (100 μL) was placed in each well of the 96-well platelet and incubated for 24 h (standard conditions). Hydrogel samples (1/10 of the well area) were prepared, placed in 5 mL of the medium and placed in the adequate wells of the plate. Importantly, we included the viability control (cell lines without tested sample, defined as K(+)), as well the cytotoxicity control (cells incubated with 1% phenol solution—a strong cytotoxicity to cells). The plates with tested samples were incubated for 24 h (standard conditions). After such an incubation, the substrate was removed and replaced with the fresh substrate (100 μL). Finally, 20 μL of the MTT reagent (concentration: 5 mg/mL; Merck, Darmstadt, Germany) was added into each well, and the plates were then incubated for 24 h (standard conditions). Next, the plates were centrifuged (parameters: 1200 rpm and 10 min), and the supernatant obtained was removed from tested cells. Then the formazan crystals were dissolved in DMSO (150 μL), the glycine buffer (25 μL) was added and the whole was incubated at ambient temperature for 15 min. Next, the liquid (100 μL) was taken from the wells, transferred to the new 96-well plate and investigated spectrophotometrically. Absorbance (at λ = 570 nm) was verified via the Spectramax multi-detection reader from Thermo Fisher Scientific (Waltham, MA, USA).

## 3. Results and Discussion

### 3.1. Investigations on the Sorption Capacity of Hydrogels

**Figure 3:**
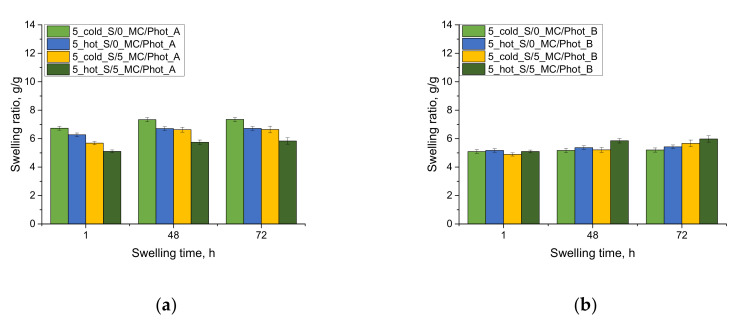

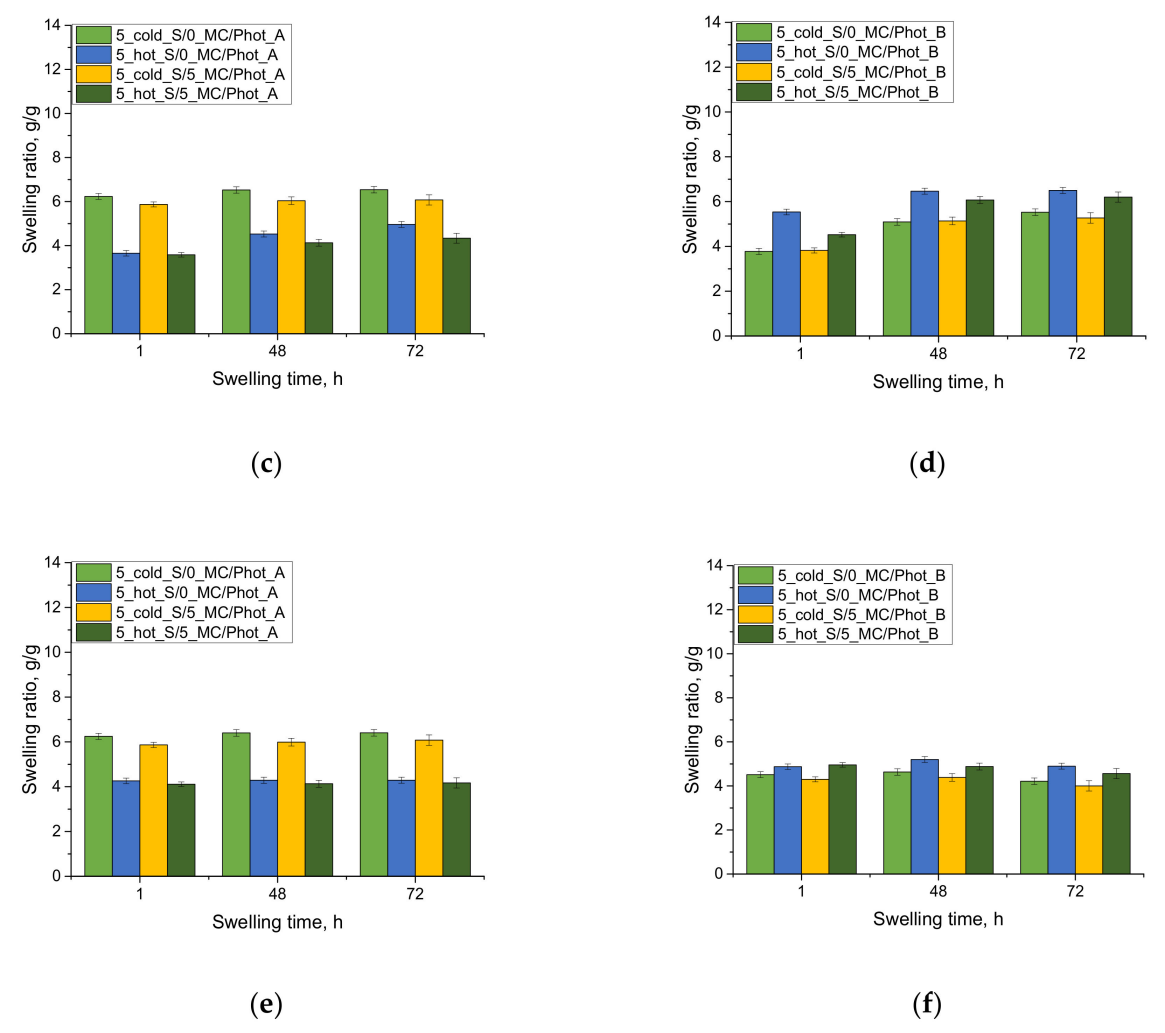
Results of swelling studies of hydrogels in distilled water (**a**,**b**), SBF (**c**,**d**) and Ringer liquid (**e**,**f**) (results for samples prepared by using photoinitiator A are presented on the left, while the results for samples prepared by using photoinitiator B are on the right).

The results of the investigations on the swelling ability of acrylic acid–based hydrogels in selected simulated physiological liquids are presented below, in Figure 3.

The first conclusion drawn based on the performed investigation was that the developed hydrogels showed swelling ability. Moreover, it was proved that hydrogels containing chamomile extract swelled to a lesser extent than materials without this additive. This results from the fact that the mentioned extract contained numerous compounds, both organic and inorganic, which may fill spaces between polymer chains in a three-dimensional polymer network, thereby reducing the sorption capacity of such modified hydrogels. It was observed that the swelling ability of all hydrogels increased rapidly during the initial swelling period. In the following swelling periods, the sorption process was much slower. The swelling kinetics of the tested hydrogels is consistent with the results presented by Budianto et al. [29], where it was demonstrated that the balance between the swelled material and the swelling media was achieved for 72 h.

Next, it was demonstrated that the polymers prepared by using photoinitiator B (phenylbis(2,4,6-trimethylbenzoyl) phosphine oxide)) exhibited a significantly lower swelling ability in distilled water compared to the materials obtained by using photoinitiator A (2-hydroxy-2-methylpropiophenone). Photoinitiator B contains, in its structure, three aromatic rings which contribute to the fact that the materials prepared by using this compound showed a more compact structure, and the polymer chains forming its matrix were characterized by limited mobility. What is more, the steric effect may also occur as a result of such structures as aromatic rings, and, in turn, absorbed liquid has a limited possibility of penetration into the tested material. On the other hand, photoinitiator A has only one aromatic ring in its structure; thus, such a phenomenon as the steric effect of limited mobility of polymer chains is not as intense as in the previous case.

In the case of the results of the studies in SBF and Ringer liquid, the impact of the type of the photoinitiator used during the synthesis on swelling properties was not as visible as in the case of the swelling in distilled water. The sorption capacity of the hydrogel samples was significantly lower in these liquids, which, in turn, is associated with the presence of numerous mono- and divalent ions in these media. Such ions may increase the crosslinking density of the hydrogels, thereby reducing their swelling ability in these liquids. The increase in the crosslinking density of hydrogels via various ions was described in Reference [30], for example.

Next, the impact of the type of the starch solution may also be observed. Higher swelling was reported for the samples prepared by using photoinitiator B and the “hot” starch than in the case of hydrogels obtained by using the same photoinitiator and the “cold” starch. This may result from the gelatinization effect [31,32]. The starch may penetrate within the polymer network, and its branched structure may reduce the availability of fluids to the spaces between polymer chains. In the case of the “hot” starch, the temperature in which the solution of this polysaccharide was prepared was higher than the temperature in which the gelatinization occurs. As a result, the molecules of amylose may be able to detach from the main chain of the starch, thus shortening the size of the entire molecule of this polysaccharide, and reduce free spaces within the polymer network, but to a lesser extent than it might take place in the case of the “cold” starch.

Moreover, the photopolymerization process performed by using photoinitiator A proceeded slower than the synthesis initiated by means of photoinitiator B. Then the starch introduced into the reaction mixture in the form of the room-temperature solution was able to integrate orderly within the hydrogel network, as a result of this photopolymerization, without significantly reducing free spaces between polymer chains available for absorbed liquid. This is the reason why hydrogels modified with “hot” starch showed higher swelling ability.

### 3.2. Assessment of the Mechanical Properties of Hydrogel Materials

**Figure 4:**
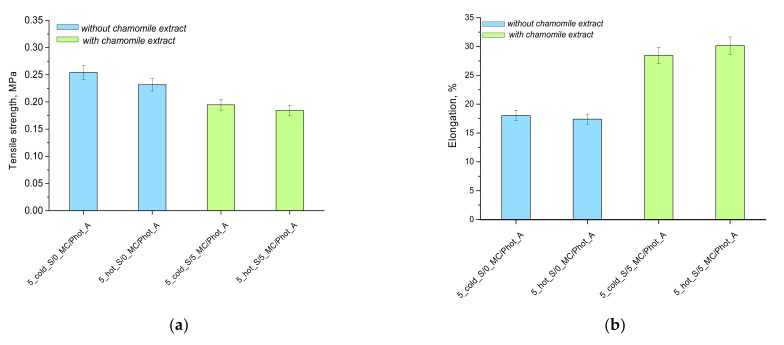
Tensile strength (**a**) and the elongation (**b**) of hydrogels under the tension applied.

The subsequent analysis involved determining the selected mechanical parameters of the obtained acrylic acid–based hydrogel matrices. Nonetheless, the study was performed only for the samples prepared by using photoinitiator A (i.e., 2-hydroxy-2-methylpropiophenone). Hydrogels obtained by means of the photoinitiator B were too fragile, thus making it impossible to prepare paddle-shaped samples. The results of the mechanical analysis are presented in Figure 4.

The samples without the chamomile extract were very hard after drying, thus making the study difficult. Such a hardness may be caused by the presence of starch, whose branched polymer chains may be able to integrate within the hydrogel polymer network, thus increasing the crosslinking density of the network and, importantly, contributing to the formation of additional crosslinks between polymer chains. On the other hand, samples containing chamomile extract showed clear elasticity. Thus, based on the research performed, it may be concluded that, in order to prepare hydrogels showing elasticity, such reagents as the starch solution at elevated temperature, as well as chamomile extract, need to be applied. Such an obtained hydrogel exhibits the most favorable mechanical properties, due to the gelatinization effect and the presence of the plant extract resulting in the loosening of the polymer network and, thus, the increase in the elasticity of such material. Beneficial mechanical properties, as well as an appropriate elasticity of hydrogels, indicate their great application potential as dressing materials. This extremely important, considering the necessity of the wound healing in places that are difficult to reach and with high mobility. The high elasticity of the developed materials enables their adequate adhesion to the wound, as this may affect the regeneration processes [33].

### 3.3. Morphological Analysis of Hydrogels via Scanning Electron Microscopy (SEM Technique)

**Figure 5:**
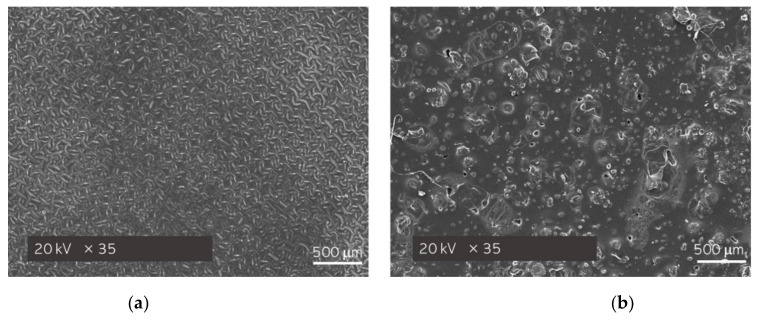
SEM images of hydrogel samples: 5_cold_S/5_MC/Phot_A (**a**) and 5_hot_S/5_MC/Phot_A (**b**).

Sample 5_hot_S/5_MC/Phot_A—the hydrogel showing the most promising mechanical properties—was additionally subjected to the surface morphology analysis via the SEM technique. Moreover, sample 5_cold_S/5_MC/Phot_A was also characterized and treated as a reference sample. The obtained SEM images are shown below, in Figure 5.

As it may be noticed, in Figure 5a, which presents the surface morphology of the hydrogel sample modified with “cold” starch, large agglomerates of unevenly distributed substance are clearly visible. On the other hand, the surface morphology of sample 5_hot_S/5_MC/Phot_A is significantly more homogeneous. Thus, it may be concluded that the hydrogel that was obtained by using the starch solution at an elevated temperature (where starch is significantly better dissolved) showed a highly uniform surface, which, undoubtedly, is its advantage.

### 3.4. Studies on the Release of the Modifying Agent from the Hydrogel Matrices

**Figure 6:**
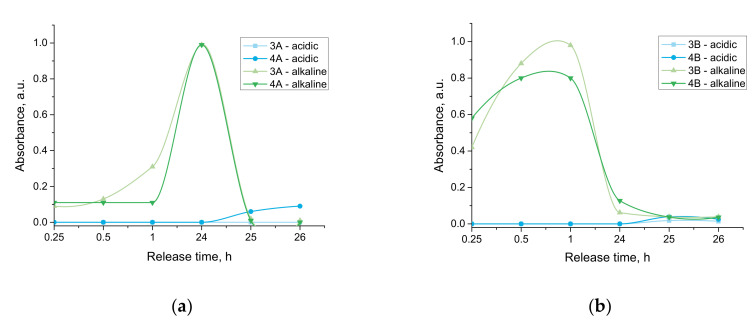
Release profile of active substance from developed hydrogels verified for samples prepared by using photoinitiator A (**a**) and photoinitiator B (**b**).

The results of the studies aimed at verifying the possibility of the release of the active substance from the developed materials, as well as determining in which environment such a release is most efficient, are presented below, in Figure 6.

Considering the above-presented results of the performed investigations, it may be concluded that the tested hydrogels showed an ability to release an active substance only in the case of the alkaline environment (PBS buffer). This is a result of the dissociation of the carboxylic group from poly(acrylic acid) into COO^−^ and H^+^ ions. Thus, in the alkaline conditions, an electrostatic repulsion of COO^−^ and OH^−^ ions takes place that, in turn, leads to the loosening of polymer chains, and, as a consequence, the release of the active substance from the polymer matrix occurs.

On the other hand, the opposite situation may be reported in acidic conditions (2% citric acid solution). In such an environment, the protonation of COO^−^ occurs; thus, the dissociation equilibrium constant shifts to the left, resulting in the hydrogel’s structure being maintained more compactly. Thus the release of the active substance practically does not occur.

Furthermore, any difference in the amount of the substance released from the materials prepared by using various photoinitiators was not observed. The only thing which may be observed involved the fact that, in the case of the samples obtained as a result of the reaction initiated by photoinitiator B (this one with more complex structure), a faster release of the active substance was reported. This is probably associated with the formation of a hydrogen bond between OH^−^ groups deriving from photoinitiator A, and OH^−^ groups present in the phosphate buffer, making the structure of the tested sample less compact.

Thus, to sum up, performed investigations provided information on the possibility of the application of developed materials as controlled drug release systems while the release process proceeds only in alkaline environments.

Carriers that are pH-responsive are an excellent solution for oral delivery systems. Based on the results obtained, it was concluded that the release of the active substance does not take place in an acidic environment. Thus, according to the literature reports, developed carriers will exhibit stability in gastric delivery of the drug and then induce controlled release in the gut (due to the alkaline pH). Such a solution may be successfully used in controlled drug release systems, significantly increasing the application potential of these materials [34].

### 3.5. Characterization of the Chemical Structure of Hydrogels via FTIR Technique

**Figure 7:**
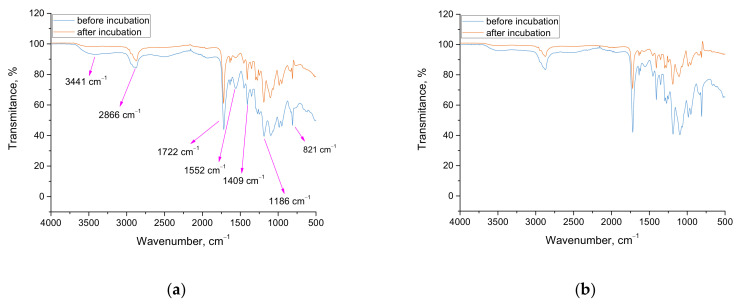
FTIR spectra of samples 5_cold_S/5_MC/Phot_A (**a**) and 5_cold_S/5_MC/Phot_B (**b**) before and after incubation in SBF.

**Figure 8:**
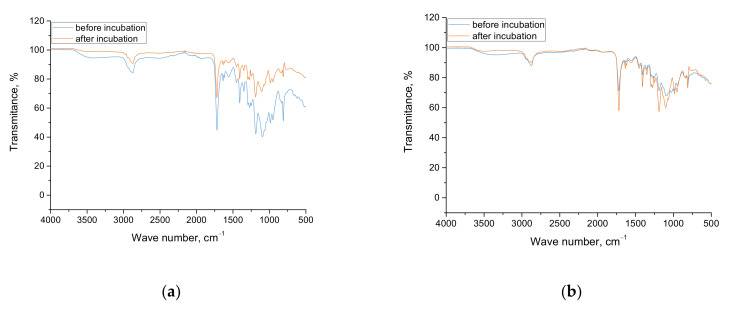
FTIR spectra of samples 5_hot_S/5_MC/Phot_A (**a**) and 5_hot_S/5_MC/Phot_B (**b**) before and after incubation in SBF.

Results of FTIR analysis are presented below, in Figure 7 and Figure 8. Importantly, the study was performed only for hydrogels modified with chamomile extract.

In Table 3, the wavenumbers visible on FTIR spectra in combination with the corresponding functional groups are presented. Additionally, the structures of both starch and acrylic acid are shown in Table 4.

When analyzing the results of the performed FTIR analysis, it may be concluded that any differences between the FTIR spectra of samples prepared by using various photoinitiators were not observed. This probably results from the fact that both photoinitiators have the same functional groups; thus, noticing the difference on the spectra of analyzed materials, as well as identifying the hydrogels prepared by using photoinitiator A or photoinitiator B, based only on their FTIR spectra is problematic.

Importantly, the results of the FTIR spectroscopy allowed to verify whether the 30-day incubation of the tested hydrogels in SBF affected their structure. As it may be observed, one from the tested materials did not degrade in such an environment, i.e., sample 5_hot_S/5_MC/Phot_B. The rest from the tested samples degraded, as is indicated by the significantly lower peak intensity visible on the spectra of hydrogels after incubation compared to the same peaks visible on the spectra of samples before the immersion in SBF. As it was mentioned, the behavior of sample 5_hot_S/5_MC/Phot_B was different—the intensity of all peaks on both FTIR spectra of this sample was the same. It was demonstrated that the hydrogel polymer obtained by using photoinitiator B (so phenylbis(2,4,6-trimethylbenzoyl) phosphine oxide) and “hot” starch showed the better stability in simulated physiological liquid. Thus, the simultaneous occurrence of the gelatinization process and the complex structure of photoinitiator B containing three aromatic rings in its structure resulted in the formation of the most stable material, which did not degrade as a result of the 30-day incubation in SBF.

Furthermore, it was observed on the FTIR spectra of 5_hot_S/5_MC/Phot_B sample that the absorption bands were more intense for that sample after incubation compared to the same absorption bands deriving from this sample before incubation. This is probably related to the leaching of the chamomile extract from hydrogel matrix. As a result, functional groups are more exposed in the hydrogel structure, which, in turn, becomes more visible from the higher intensity of the absorption bands derived from these groups. On the other hand, as it was previously mentioned, there is no degradation of this sample, but the release of the active substance from its interior takes place.

Importantly, Wu et al. proved a quantitative correlation between the release of the active substance and the degradation of hydrogel materials. Thus, it may be concluded that, in the case of materials which degrade, the release of the active substances will be much faster than in the case of sample 5_hot_S/5_MC/Phot_B, as it does not show degradation [35].

### 3.6. Evaluation of the Thermal Properties of Hydrogels

**Figure 9:**
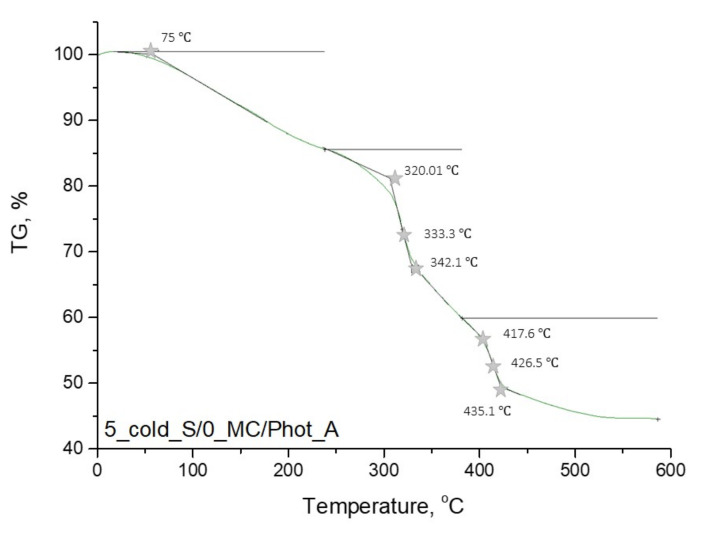
Thermogravimetric analysis of hydrogel sample: 5_cold_S/0_MC/Phot_A.

**Figure 10:**
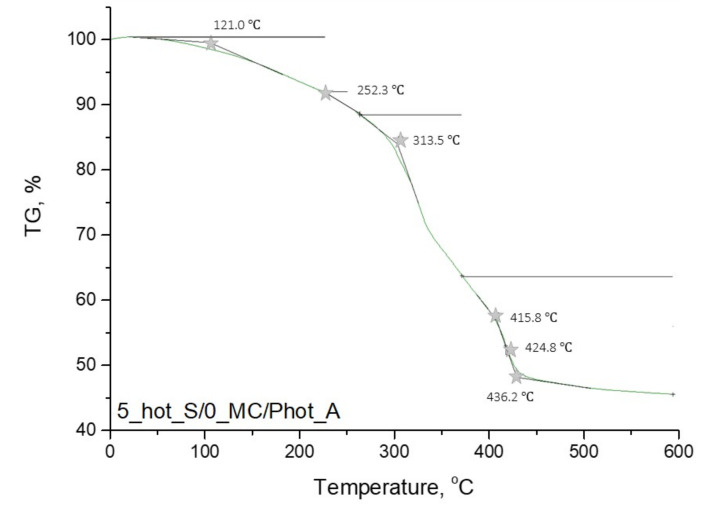
Thermogravimetric analysis of hydrogel sample: 5_hot_S/0_MC/Phot_A.

**Figure 11:**
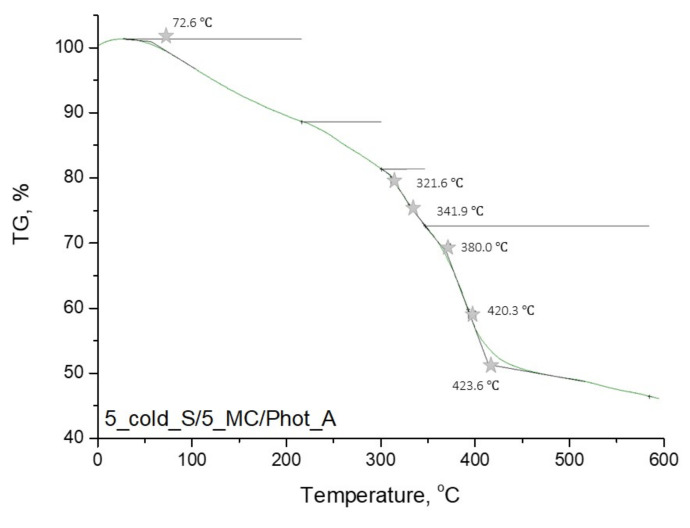
Thermogravimetric analysis of hydrogel sample: 5_cold_S/5_MC/Phot_A.

**Figure 12:**
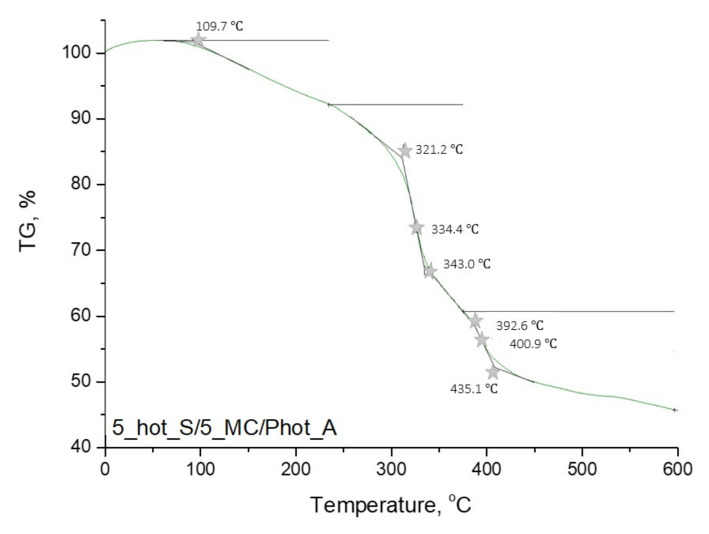
Thermogravimetric analysis of hydrogel sample: 5_hot_S/5_MC/Phot_A.

In Figure 9, Figure 10, Figure 11 and Figure 12, the results of the performed thermogravimetric analysis are presented.

The performed thermogravimetric analysis of the developed hydrogels showed that the form of the starch introduced into the hydrogel matrix, as well as the presence of *Matricaria chamomilla* L. extract, affected the thermal stability of the analyzed materials. The samples prepared by using “hot” starch were characterized by better thermal stability than these ones containing “cold” starch. Additionally, it was demonstrated that the introduction of the mentioned extract of plant origin resulted in the slight decrease of the thermal stability of these materials regardless of the form of starch which was used for the hydrogels’ modification.

The first loss of mass results from the loss of moisture by the tested hydrogel samples. In the next step, intra- and intermolecular anhydride rings are formed. This step begins within the temperature range of 250–270 °C. Further mass loss results from the decarboxylation process, which takes place at a temperature of about 320 °C. Then the decomposition of the anhydride rings, proceeding with the release of CO_2_, takes place. As a result, such compounds as ketenes, ketones or alcohols are formed. Next, above 400 °C, the remaining anhydride rings decompose, leading to the release of numerous volatile compounds. The thermal decomposition of starch present in the tested material consists of two stages. At the beginning (at a temperature of about 100 °C), water and other volatile compounds evaporate. The main thermal degradation process begins around 300 °C. On the TGA curves obtained, a certain mass loss of the tested sample was observed within the temperature range of about 330–340 °C. These temperature values differ slightly from the literature values. This is due to the fact that this value applies to pure starch, while, in this case, this polysaccharide constitutes a component of a multicomponent polymer material [36,37,38].

### 3.7. Evaluation of Cytotoxicity of Developed Hydrogels via MTT Reduction Assay

**Figure 13:**
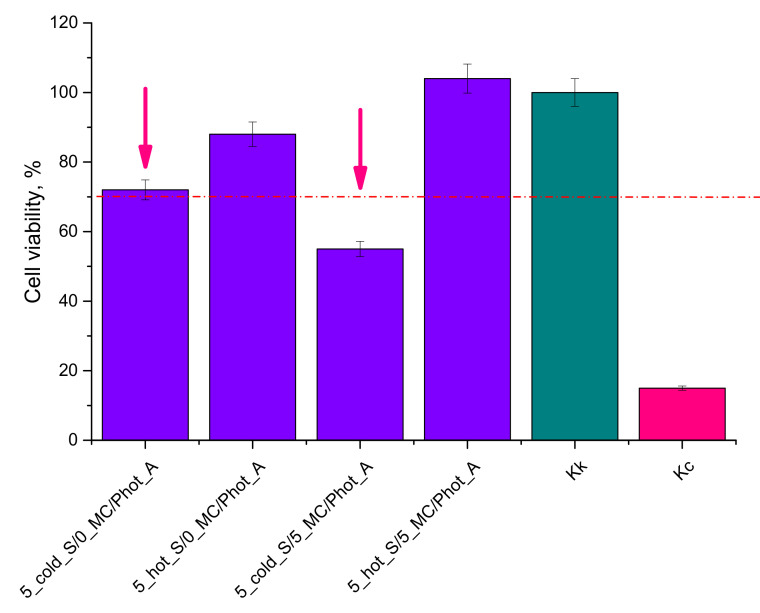
Results of cytotoxicity analysis of hydrogels via MTT reduction assay (the red doted line indicates the survival limit of the cells below which the material is defined as cytotoxic).

In Figure 13, the results of the performed studies on the cytotoxicity of the developed materials are presented.

In Figure 13 the viability of cells incubated in the presence of the hydrogels has been presented while in parallel K_k_ control (negative control; cells in the culture without tested hydrogel) and K_c_ control (positive control; cells treated with 1% phenol solution showing cytotoxicity towards them) have been performed.

According to the ISO standard, the biomaterial is not considered as cytotoxic when the viability of cells incubated for 24 h in its presence of is over 70% [39]. Thus, based on the results of the performed investigations, it may be reported that hydrogels modified with “hot” starch showed no cytotoxicity toward L929 murine fibroblasts, while the modifier in a form of *Matricaria chamomilla* L. extract additionally affected the proliferation of tested cell lines. In turn, hydrogels modified with “cold” starch were characterized by cytotoxicity to the analyzed cells, while the presence of *Matricaria chamomilla* L. extract increased the cytotoxic effect. Thus, it may be assumed that the photopolymerization process did not run properly in this case, resulting in the insufficient crosslinking of such obtained materials. As a consequence, a cytotoxicity of these hydrogels was observed. Moreover, introduction of the *Matricaria chamomilla* L. extract into the reaction mixture resulted in its dilution. This, in turn, affected the photopolymerization process. Finally, during the incubation of such prepared materials with tested cell lines, a release of unreacted reagents compounds (including acrylic acid, potassium hydroxide or crosslinking agent) from the interior of insufficiently crosslinked hydrogel matrix took place. These compounds may be toxic to the tested cells, thus affecting their viability. Therefore, the performed investigations allowed us to conclude that only hydrogel materials modified with “hot” starch may be considered for further, more advanced biological analyses in terms of their application potential as innovative dressing materials.

## 4. Conclusions

- The type of the photoinitiator used during the synthesis, the type of the starch solution (temperature of its preparation) and the modifier in the form of *Matricaria chamomilla* L. extract affected the physicochemical properties of hydrogels.
- The hydrogels modified with *Matricaria chamomilla* L. extract showed a slightly lower sorption capacity than unmodified polymers. This may result from the presence of numerus organic and inorganic compounds in the extract that may fill free spaces within polymer network.
- The hydrogels prepared by using phenylbis(2,4,6-trimethylbenzoyl) phosphine oxide as a photoinitiator showed lower swelling ability than hydrogels obtained as a result of the photopolymerization initiated by 2-hydroxy-2-methylpropiophenone. This may be caused by the presence of aromatic rings in the structure of phenylbis(2,4,6-trimethylbenzoyl) phosphine oxide, which limits the penetration of absorbed liquid within the polymer network via the so-called steric effect.
- The release of the active substance (i.e., chamomile extract) from the developed hydrogels proceeded only in an alkaline environment.
- Over 90% of chamomile extract was released in the alkaline environment from hydrogels prepared by using phenylbis(2,4,6-trimethylbenzoyl) phosphine oxide as a photoinitiator. Polymers obtained by means of 2-hydroxy-2-methylpropiophenone needed 24 h for the release of such an amount of the active substance.
- Among the hydrogels modified with chamomile extract, the sample prepared by using phenylbis(2,4,6-trimethylbenzoyl) phosphine oxide as a photoinitiator and the starch solution at elevated temperature as a modifier showed the highest stability in the simulated physiological liquid. This was probably caused by the starch gelatinization effect and the complex structure of applied photoinitiator. The rest of analyzed hydrogels degraded in such an environment.
- For the hydrogel obtained by using 2-hydroxy-2-methylpropiophenone as a photoinitiator, the starch solution at the elevated temperature and chamomile extract showed the highest elasticity, as well as the most homogeneous surface morphology.
- The choice of the most beneficial hydrogel composition is strongly influenced by its intended application. For example, the sample showing the highest elasticity may be used as a dressing material. On the other hand, hydrogels which did not degrade as a result of the 30-day incubation in simulated physiological liquid may be used as elements of implants intended for tissue regeneration requiring a longer time to fix. In turn, an easily degradable sample may be applied as a drug carrier which is ultimately biodegradable in the body.
- The hydrogel materials modified with “hot” starch showed higher thermal stability than hydrogels containing “cold” starch. Moreover, the introduction of *Matricaria chamomilla* L. extract into the hydrogel matrices affects the deterioration of their thermal stability. Nonetheless, in terms of the potential application of developed hydrogels as innovative III generation dressings or drug delivery systems, such a result will not disqualify these materials for the abovementioned applications.
- The hydrogel materials modified with “hot” starch showed no cytotoxicity toward L929 murine fibroblasts. Importantly, hydrogels containing *Matricaria chamomilla* L. extract increase the cell viability by 15%, thus indicating the cell proliferation. Materials modified with “cold” starch demonstrated cytotoxicity toward tested cell lines; therefore, they cannot be applied for biomedical purposes.
- All of the developed hydrogels showed great application potential and may be successfully widely considered to be applied for biomedical purposes; therefore, in the nearest future, we plan to subject them to more advanced in vitro and in vivo biological analyses.

## Figures and Tables

**Table 1:** Compositions of acrylic acid–based hydrogels.

Sample	Acrylic Acid, mL	40% KOH Solution, mL	5% “Cold” Starch *v*/*v*, mL	5% “hot” starch *v*/*v*, mL	*Matricaria chamomilla* L. Extract, mL	Crosslinker *, mL	Photoinitiator A **, mL	Photoinitiator B ***, mL
1A	15	17	5	-	-	5	0.25	-
2A			-	5	-			
3A			5	-	5			
4A			-	5	5			
1B			5	-	-		-	0.25
2B			-	5	-			
3B			5	-	5			
4B			-	5	5			

* Diacrylate poly(ethylene glycol) M_n_ = 575 g/mol. ** 2-hydroxy-2-methylpropiophenone, Darocur 1173. *** Phenylbis(2,4,6-trimethylbenzoyl) phosphine oxide.

**Table 2:** Name of compositions of acrylic acid–based hydrogels.

Sample	Name of Sample
1A	5_cold_S/0_MC/Phot_A
2A	5_hot_S/0_MC/Phot_A
3A	5_cold_S/5_MC/Phot_A
4A	5_hot_S/5_MC/Phot_A
1B	5_cold_S/0_MC/Phot_B
2B	5_hot_S/0_MC/Phot_B
3B	5_cold_S/5_MC/Phot_B
4B	5_hot_S/5_MC/Phot_B

**Table 3:** Wavenumbers and the corresponding functional groups.

Wavenumber, cm^−1^	Functional Group
3441	O-H
2866	C-H aliphatic
1722	C=O acidic
1552	C=C aromatic
1409	O-H
1186	C-O
821	C-H

**Table 4:** Structural formulas of starch and acrylic acid.

No.	Compound	Structural Formula
1.	Starch	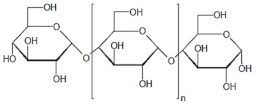
2.	Acrylic acid	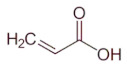

## Data Availability

The data presented in this study are available upon request from the corresponding authors.
